# Appraisal of novel azomethine–thioxoimidazolidinone conjugates as ecto-5′-nucleotidase inhibitors: synthesis and molecular docking studies†

**DOI:** 10.1039/d2ra02675a

**Published:** 2022-06-14

**Authors:** Pervaiz Ali Channar, Sehrish Bano, Sidra Hassan, Fouzia Perveen, Aamer Saeed, Parvez Ali Mahesar, Imtiaz Ali Khan, Jamshed Iqbal

**Affiliations:** Department of Chemistry, Quaid-I-Azam University Islamabad 45320 Pakistan aamersaeed@yahoo.com asaeed@qau.edu.pk; Department of Basic Sciences, Mathematics and Humanities, Dawood University of Engineering and Technology Karachi 74800 Pakistan; Centre for Advanced Drug Research, COMSATS University Islamabad, Abbottabad Campus Abbottabad-22060 Pakistan drjamshed@cuiatd.edu.pk; Research Center for Modeling and Simulations, National University of Sciences and Technology (NUST) Islamabad Pakistan; Institute of Chemistry, Shah Abdul Latif University Khairpur 66020 Pakistan; Department of Entomology, Agricultural University Peshawar 25130 Khyber Pakhtunkhwa Pakistan

## Abstract

Purinergic signaling is regulated by a group of extracellular enzymes called ectonucleotidases. One of its members *i.e.*, ecto-5′-nucleotidase (*h*-e5′NT) is involved in the final step of the enzymatic hydrolysis cascade that is the conversion of adenosine monophosphate (AMP) to adenosine and therefore, involves the regulation of adenosine level in extracellular space. The overexpression of *h*-e5′NT has been observed in various pathological conditions such as hypoxia, inflammation and cancers, and led to various complications. Hence, the identification of a potent as well as selective inhibitor of *h*-e5′NT is of greater importance in therapeutic treatment of various diseases. Azomethine-thioxoimidazolidinone derivatives were studied for their inhibition potential against e5′NT enzyme along with cytotoxic potential against cancer cell lines possessing overexpression of e5′NT enzyme. The derivative (*E*)-3-((4-((3-methoxybenzyl)oxy)benzylidene)amino)-2-thioxoimidazolidin-4-one (4g) displayed selective and significant inhibition towards *h*-e5′NT with an IC_50_ value of 0.23 ± 0.08 μM. While two other derivatives *i.e.*, (*E*)-3-(((5-bromothiophen-2-yl)methylene)amino)-2-thioxoimidazolidin-4-one (4b) and 2-thioxo-3-((3,4,5-trimethoxybenzylidene)amino)imidazolidin-4-one (4e), exhibited non-selective potent inhibitory behavior against both human and rat enzymes. Moreover, these derivatives (4b, 4e and 4g) were further investigated for their effect on the expression of *h*-e5′NT using quantitative real time polymerase chain reaction. Additionally, molecular docking and DFT studies were also performed to determine the putative binding mode of potent inhibitors within the enzyme active site. HOMO, LUMO, Δ*E*, and molecular electrostatic potential maps were computed by DFT and the charge transfer regions within the molecules were identified to find out the regions for electrophilic and nucleophilic attack.

## Abstract 2

Azomethine–thioxoimidazolidinone conjugates as ecto-5′-nucleotidase inhibitors.

## Graphical abstract

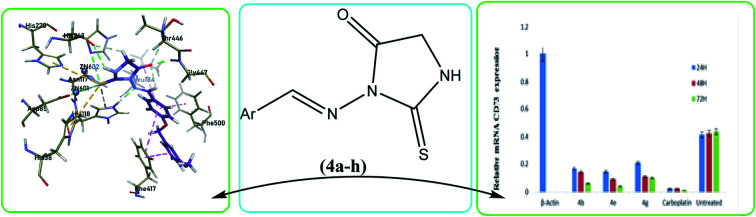

## 1. Introduction

Ecto-5′NT is a metallophosphatase enzyme bearing two zinc atoms in its active site. It is glycophosphatidyl anchored extracellular enzyme that works along with NTPDase in catabolism of nucleotide molecules; NTPDase 1 isozyme catalyzes the dephosphorylation of ATP into AMP whereas CD73 takes part in the hydrolysis of AMP into adenosine.^1^ In addition, e5′NT is also involved in a non-enzymatic role such as cell type-specific functions including epithelial cell transport and tissue barrier function,^2,3^ inhibition of endothelial permeability,^4^ reinforcement of lymphocyte–endothelium interactions,^5^ inhibition of macrophage and mesenchymal cell-mediated inflammation,^6,7^ hyperpolarization and relaxation of smooth muscle cells, and antinociception *via* modulation of neuronal activity.^8^

It has been found that the hypoxic conditions of tumor microenvironment results in increased extracellular expression of both ectonucleotidase enzymes *i.e.*, CD39 and CD73, ultimately increases the production of adenosine.^9^ In tumor microenvironment, the overexpressed adenosine plays an important role in tumor immunoescape mechanism regardless of other immune modulating factors. Additionally, it also increases the tumor angiogenesis, growth and metastasis. Its role has been studied in several carcinomas, for example, colorectal, breast, bladder, pancreas, ovarian, leukemia and melanoma.^10^ Among them cervical cancer is highly prevalent among women as the fourth major cause of fatality rate worldwide. The cervical cancer is majorly caused by the infection of human papilloma virus (HPV). The defined role of CD73 in cervical cancer cells has not been fully established.^11–13^ Therefore, CD73 acting as a regulator of adenosine production, has been treated as valuable drug target in several conditions such as autoimmunity, cancer, ischemic-reperfusion injury and allergy. Previous studies have exhibited the importance of CD73 inhibition by using a substrate analogue APCP or a monoclonal antibody with resultant reduction in tumor growth and metastasis. Hence, CD73 has been regarded as a potential therapeutic target to establish new anticancer therapy. In view of pharmacological role of e5'NT enzyme in tumor development, an effort is carried out to find out the potential therapeutical inhibitors with selectivity as well as greater potency.

Here in present study, we try to synthesize a novel CD73 inhibitor with greater activity against the target enzyme. The basic pharmacophore *i.e.*, 2-thioxoimidazolidinone molecule is used to prepare a hybrid conjugate; azomethine-2-thioxoimidazolidinones. 2-Thioxoimidazolidinones, commonly known as thiohydantoins, are sulfur analogs of hydantoins (2,4-imidazolidinediones) in which one or both carbonyl groups have been substituted with thiocarbonyl groups. By adding groups to provide steric bulk, more hydrophilic or hydrophobic contacts, or stacking, the backbone of thiohydantoin may be readily changed to adopt the preferred structural type over another. Previous literature established the importance of thioxoimidazolidinones in medicinal chemistry as an antitumor, antischistosomiatic, anti-inflammatory, hypoglycemic, antiviral, antitubercular, analgesic, anticonvulsant, antimicrobial, and antifungal agents.^14–22^ It has not been previously used or tested against CD73 target. Additionally, 3,5-disubstituted-2-thioxoimidazolidinones and their glycoside derivatives displayed higher activity against herpes simplex (HSV) and human immunodeficiency viruses (HIV), leukemia, and prostate cancer.^23,24^ Combination of two or more bioactive nuclei is considered as a clever approach in drug designing. Azomethines are widely known as Schiff bases; which represent a pharmacologically active class of organic compounds. Herein, we have coupled medicinally active 2-thioxoimidazolidinones with azomethine to seek their biological potential as azomethines were previously reported as ecto-5′-nucleotidase inhibitors. The compounds containing azomethine linkage (C=N) such as imines, hydrazones, and oximes are extremely valuable compounds in the field of synthetic organic chemistry.

## 2. Experimental

### 2.1 General procedure for the synthesis of ((aryl) methylene) hydrazine-1-carbothioamides (3a–h)

To a stirred solution of thiosemicarbazide (1) (1.0 mM) in 25 mL absolute ethanol, suitably substituted aldehyde, or ketone (2) (1.0 mM) along with 1–2 drops of conc. Sulfuric acid were added and the mixture was refluxed for 12 h. On completion indicated by TLC, the reaction mixture was cooled to room temperature, and the solids obtained were filtered and recrystallized from ethanol to give compounds (3a–h).

#### 2.1.1 General procedure for the synthesis of ((*E*)-3-(aryl)amino)-2-thioxoimidazolidin-4-one (4a–h)

A mixture of (3a–h) (0.01 mol), ethyl chloroacetate (0.01 mol), in ethanol (50 mL) in presence of fused sodium acetate (0.03 mol) was heated under reflux for 6 h, then cooled to room temperature and poured into ice-water. The solid formed was filtered off, dried and purified by suitable solvents to afford (4a–h).

##### 2.1.1.1 (*E*)-3-((4-Chloro-3-nitrobenzylidene)amino)-2-thioxoimidazolidin-4-one (4a)

Yield: 72%; mp 285 °C, *R*_f_ = 0.51 IR; (KBr, cm^−1^): 3292 (N–H), 3154 (sp^2^CH), 1570 (C=N), 1560 (Ar–C=C), ^1^H NMR: (DMSO-d_6_, 300 MHz): 11.60 (s, 1H, NH), 8.43 (s, 1H, CH=N), 7.54 (d, 2H, *J* = 8.1 Hz, Ar–H), 7.4 (s, 1H), 3.98 (S, 2H, CH_2_N) ^13^C NMR (75 MHz, DMSO-*d*_6_): *δ* (ppm): 178.5, (C=S) 173.1, (C=O), 154.5, (CH=N) 147.7, 143.3, 141.5, 139.5, 137.5, 129.9, 56.9. Anal. calcd for C_10_H_7_ClN_4_O_3_S C, 40.21; H, 2.36; N, 18.76; S, 10.73 found C, 40.20; H, 2.37; N, 18.74; S, 10.75 found: 392.

##### 2.1.1.2 (*E*)-3-(((5-Bromothiophen-2-yl)methylene)amino)-2-thioxoimidazolidin-4-one (4b)

Yield: 75%; mp 222 °C, *R*_f_ = 0.66 IR; (KBr, cm^−1^): 3251 (N–H), 3162 (sp^2^CH), 1652 (C=N), 1630 (Ar–C=C), ^1^H NMR: (DMSO-d_6_, 300 MHz): 11.94 (s, 1H, NH), 8.41 (s, 1H, CH=N), 7.32 (dd, 1H), 7.20 (dd, 1H), 3.91 (S, 2H, CH_2_N) ^13^C NMR (75 MHz, DMSO-d_6_): 174.4, 165.7, 150.2, 141.3, 132.7, 131.9, 116.09, 33.5. Anal. calcd for C_8_H_6_BrN_3_OS_2_C, 31.59; H, 1.99; N, 13.81; S, 21.08 found: C, 31.57; H, 1.98; N, 13.80; S, 21.06 found: 302.

##### 2.1.1.3 (*E*)-3-(((5-Methylfuran-2-yl)methylene)amino)-2-thioxoimidazolidin-4-one (4c)

Yield: (75%): mp 240 °C *R*_f_: 0.42; IR; (KBr, cm^−1^): 3414 (NH_2_), 3262 (N–H), 3123 (sp^2^CH), 2932 (sp^3^CH), 1601 (C=N), 1585 (Ar–C=C), ^1^H NMR; (300 MHz, DMSO): *δ* 10.52 (s, 1H, NH), 7.53 (s, 1H, CH=N), 6.18 (dd, 1H), 7.6 (dd, 1H), 3.70 (S, 2H, CH_2_N) 2.20 (s, 3H). ^13^C NMR (75 MHz): *δ* 181.1, (C=S) 170.2, (C=O), 148.5, (CH=N) 155.76, 147.9, 108.7, 110.5, 56.1, 14.4 anal. calcd for C_9_H_9_N_3_O_2_S C, 48.42; H, 4.06; N, 18.82; S, 14.36 found: C, 48.40; H, 4.08; N, 18.81; S, 14.37 found: 223.

##### 2.1.1.4 (*E*)-3-(((9,10-Dihydropyren-4-yl)methylene)amino)-2-thioxoimidazolidin-4-one (4d)

Yield: (80%), mp 205 °C; *R*_f_: 0.51; IR; (KBr, cm^−1^): 3223 (N–H), 1600 (C=N), 1583 (Ar–C=C), 1082 (C=S), cm^−1^; ^1^H NMR: (DMSO-d6, 300 MHz): *δ*: 10.42 (s, 1H, NH), 8.2–8.5 (m, 3H, Ar–H), 7.97 (s, 1H, CH=N), 7.94–7.92 (m, 2H, Ar–H), 7.7–7.62 (m, 4H, Ar–H), 3.77 (S, 2H, CH_2_N) ^13^C NMR (75 MHz) *δ*: 184.0 (C=S), 171.2, (C=O), 141 (C=N), 127, 130, 132, 133, 134, 137, 140, 143, 14558.1, calcd for C_20_H_15_N_3_OS: C, 69.54; H, 4.38; N, 12.17; S, 9.28 found: C, 69.56; H, 4.36; N, 12.19; S, 9.26 found: 345.

##### 2.1.1.5 (*E*)-2-Thioxo-3-((3,4,5-trimethoxybenzylidene)amino)imidazolidin-4-one (4e)

Yield: (70%), mp 215 °C; *R*_f_: 0.55; IR; (KBr, cm^−1^): 3220 (N–H), 1605 (C=N), 1571 (Ar–C=C), 1080 (C=S), cm^−1^; ^1^H NMR: (DMSO-d6, 300 MHz): *δ*: 11.96 (s, 1H, NH), 8.32 (s, 1H, CH=N), 7.09 (s, 2H, Ar–H), 3.83–3.81 (S, 9H, CH_3_O)3.88 (S, 2H, CH_2_N) ^13^C NMR (75 MHz) *δ*: 174.6 (C=S), 165.4, (C=O), 153.5, 140.1 (C=N), 130.6, 105.3, 60.6, 56.5, 56.3, 33.4, calcd for C_13_H_15_N_3_O_4_S: C, 50.47; H, 4.89; N, 13.58; S, 10.37: found C, 50.47; H, 4.89; N, 13.58; S, 10.37 found: 309.

##### 2.1.1.6 (*E*)-3-((4-(Benzyloxy)benzylidene)amino)-2-thioxoimidazolidin-4-one (4f)

Yield: 72%; solid mp 265 °C, *R*_f_ = 0.61 IR; (KBr, cm^−1^): 3223 (N–H), 3120 (sp^2^CH), 2964 (sp^3^CH), 1605 (C=N), 1593 (Ar–C=C), cm^−1 1^H NMR: (DMSO-d6, 300 MHz): 11.88 (1H, s, broad, NH), 8.0 (s, 1H, HC=N), 7.40 (d, 5.4 Hz, 2H), 7.30 (m, 5H), 7.12 (d, 2H, *J* = 8.1 Hz, Ar–H), 5.11 (s, 2H, OCH_2_); 3.57 (S, 2H, CH_2_N) ^13^C NMR (75 MHz, DMSO-d_6_): 177.6, (C=S), 171.2, (C=O), 161.5, 150.1, (C=N), 143.2, 136.5, 132.2, 131.0, 128.7, 127.5, 114.2, 70.7, 58.3 anal. calcd for C_17_H_15_N_3_O_2_S: C, 62.75; H, 4.65; N, 12.91; S, 9.85 found: C, 62.73; H, 4.62; N, 12.90; S, 9.83 found: 325.

##### 2.1.1.7 (*E*)-3-((4-((3-Methoxybenzyl)oxy)benzylidene)amino)-2-thioxoimidazolidin-4-one (4g)

Yield: 82%; solid mp 280 °C, *R*_f_ = 0.61 IR; (KBr, cm^−1^): 3220 (N–H), 3122 (sp^2^CH), 2961 (sp^3^CH), 1600 (C=N), 1583 (Ar–C=C), cm^−1^; ^1^H NMR: (DMSO-d6, 300 MHz): 12.20 (s, 1H, NH), 8.02 (s, 1H, HC=N), 7.80 (d, 6.3 Hz, 2H), 7.60 (6.3 Hz, 2H) 7.4–67.1 (m, 4H), 5.12 (s, 2H, OCH_2_); 3.90 (s, 3H, OCH_3_); 3.47 (S, 2H, CH_2_N) ^13^C NMR (75 MHz, DMSO-d_6_): 178.9, (C=S), 172.2 (C=O), 161.6, 160.8, 153.5, (C=N), 143.8, 132.1, 128.9, 127.3, 122.0, 120.0, 113.9, 109.3, 72.4; 55.9, 55.4. Anal. calcd for C_18_H_17_N_3_O_3_S: C, 60.83; H, 4.82; N, 11.82; S, 9.02 found C, 60.81; H, 4.84; N, 11.80; S, 9.04 found: 355.

##### 2.1.1.8 (*E*)-3-((Pyridin-3-ylmethylene)amino)-2-thioxoimidazolidin-4-one (4h)

Yield: 61%; solid mp 268 °C, *R*_f_ = 0.61 IR; (KBr, cm^−1^): 3231 (N–H), 3132 (sp^2^CH), 1605 (C=N), 1581 (Ar–C=C), cm^−1 1^H NMR: (DMSO-d6, 300 MHz): 12.00 (s, 1H, NH), 8.88 (dd, *J* = 7.5, 1.3 Hz, 1H), 8.61 (d, *J* = 1.3 Hz, 1H), 8.46 (dt, *J* = 7.5, 1.4 Hz, 1H), 8.11 (s, 1H, HC=N), 7.48 (t, *J* = 7.5 Hz, 1H), 3.90 (S, 2H, CH_2_N) ^13^C NMR (75 MHz, DMSO-d_6_): 174.7 (C=S), 167.0, (C=O), 154.1, (C=N), 151.6, 149.6, 134.4, 130.5, 124.4, 33.5 Anal. calcd for C_9_H_8_N_4_OS: C, 49.08; H, 3.66; N, 25.44; S, 14.56 found: C, 49.08; H, 3.66; N, 25.40; S, 14.58 found: 220.

### 2.2 Biochemical assays

#### 2.2.1 Cell transfection with ecto-5′-nucleotidase

The plasmid expressing both rat and human e-5′NT were employed to transfect the COS-7 cells *via* lipofectamine. The cells after becoming confluent, were incubated at 37 °C in growth media *i.e*., DMEM/F-12 having 6ug plasmid DNA and 24 μL transfecting reagent. After 5 h, the transfection was stopped by replacing the same volume of media containing only 10% FBS. Finally the transfected cells were collected from the media after 48–72 h.

##### 2.2.1.1 Preparation of membrane fractions

The confluent transfected cells were collected from the harvesting buffer through scraping and subsequently washed with Tris-saline buffer along with centrifugation by spinning for 5 min at 300 × *g* 4 °C.^25^ The obtained cell pellet was allowed to remain suspended in the harvesting buffer with aprotinin (10 μg mL^−1^) and sonicated. The cells were again centrifuged for 10 min at 800 × *g* 4 °C to remove the cellular and nuclear debris. Finally, glycerol (7.5%) was incorporated to the resultant supernatant. Bradford microplate assay^26^ was used for protein quantification employing Bovine serum albumin as standard reference.

##### 2.2.1.2 Ecto-5′-nucleotidase inhibition assay

The ecto-5′-nucleotidase inhibition assay was carried out by using P/ACE MDQ capillary electrophoresis system (Beckman Instruments, Fullerton, CA, USA) according to previously reported method.^27^ The compound solutions were prepared and analyzed at 0.1 mM concentration in assay buffer (1 mM CaCl_2_, 10 mM Tris HCl and 2 mM MgCl_2_, pH 7.4). The 10 μL of each sample was used in assay volume of 100 μL along with 10 μL of *h*-e5′NT (6.94 μg mL^−1^) protein extract or *r*-e5′NT (7.17 μg mL^−1^) and 70 μL of assay buffer. After 10 min incubation at 37 °C, 10 μL of substrate AMP (adenosine monophosphate) at final concentration of 500 μM was added to start the enzymatic reaction. After 30 min incubation at 37 °C, the reaction was stopped *via* thermal denaturation by heating the reaction mixture in water bath at 99 °C for 20 min. Finally, 50 μL of this reaction mixture was used for analysis. It was hydrodynamically injected into the capillary under pressure of 0.5 psi for 5 s and 15 kV voltage to allow the separation of substrate and product peaks. The compounds with more than 50% inhibitory activity of either human or rat enzyme were further evaluated for determination of IC_50_ values. For this purpose, serial dilutions of each active compound were prepared and analyzed to obtain the dose response curve by using the above protocol. All experiments were performed in triplicate. The non-linear regression analysis of program PRISM 5.0 (GraphPad, San Diego, California, USA) was used to calculate the IC_50_ values.

#### 2.2.2 Cell lines and cell cultures

HeLa cells and BHK-21 cells were cultured in flasks containing DMEM medium along with penicillin (100 U mL^−1^), streptomycin (100 μg mL^−1^), amphotericin B (10 μg mL^−1^) and heat-inactivated FBS (10%). These cell lines were incubated at 37 °C in 5% CO_2_ incubator until became confluent. In the end, the confluent monolayer cells (1 × 10^4^ cells per mL) were seeded in 96-well plate to conduct initial cytotoxicity experiments.

##### 2.2.2.1 Cell viability assays (MTT assay)

The cytotoxic potential of azomethine–thioxoimidazolidine conjugates was carried out according to the previously reported method by employing MTT (Dimethyl-2-thiazolyl-2,5-diphenyl-2*H*-tetrazolium bromide) reagent.^28,29^ All the compounds were analyzed against cervical cancer (HeLa). Accordingly, 90 μL of the cell culture (2.5 × 10^4^ cells per mL cells) was seeded/well for overnight in 96 well plates. Then the cells were treated with 10 μL of respective test compounds (at final concentration of 100 μM) and plates were incubated at 37 °C for 24 h in CO_2_ incubator. Negative control (100 μL culture medium) along with positive (100 μM of carboplatin) control was also examined with the tested compounds. After incubations, 10 μL of MTT reagent (5 mg mL^−1^) was added to each well and plates were again kept at 37 °C for 4 h. Subsequently 100 μL of stopping reagent (50% isopropanol and 50 mL of 10% sodium dodecyl sulfate) was added to stop the enzyme reaction and solubilized the formazan crystals. The plates were allowed to incubate at room temperature for 30 min under agitation. Finally, absorbance was measured using a 96 well microplate reader (Bio-Tek ELx 800™, Instruments, Inc. Winooski, VT, USA) at 570 nm subtracting the background absorbance at 690 nm. All experiments were carried out in triplicate. Results were reported as mean of three independent experiments (±SEM) and expressed as percent inhibitions.

#### 2.2.3 Determination of mRNA expression of CD73 using real time-PCR

CD73 mRNA expression was analyzed by real-time PCR in which total volume of 20 μL of reaction buffer containing 1 μL cDNA, 0.25 μL CD73 forward primer (10 pmol μL^−1^), 0.25 μL CD73 reverse primer (10 pmol μL^−1^), 10 μL of SYBR green master mix (WizPURE™) and Ultra-pure water 8.5 μL was used. Same reaction buffer was prepared for housekeeping gene β-actin. The PCR cycling conditions were as follows: 5 min at 95 °C, 45 s at 60 °C for 40 cycles. CD73 mRNA expression was determined as the ratio of the CD73 to a housekeeping gene β-actin.^30^

#### 2.2.4 Molecular docking studies

Molecular docking studies were carried out to analyze the putative binding mode of all compounds within the active site of target enzymes *i.e.*, *h*-e5′NT and *r*-e5′NT. X-ray crystallographic structures of *h*-e5′NT protein were downloaded from the RCSB Protein Data Bank in the form of PDB ID: 4H2I^31^ whereas due to unavailability of X-ray crystallographic structure of *r*-e5′NT, previously reported model was employed.^32^ First 2D structures of all compounds were created using Marvin Sketch of ChemAxon suit academic licensed^33^ and then converted into 3D structure *via* Molecular Builder program implemented in Molecular Operating Environment (MOE 2019.0201).^34^ The crystal structure of *h*-e5′NT revealed the presence of unwanted water along with other co-crystalized ligands within the active sites, which were removed before docking calculation. Initially, both target enzymes as well as compounds were protonated with energy minimized up to 0.05 gradient *via* MMFF94x force field using Protonate 3D tool of MOE 2019.0201. MOE-Dock tool in MOE 2019.0201 was used for molecular docking calculation of selected compound with target enzymes.^34^ Against *h*-e5′NT, active site was selected around the co-crystallized ligands while in case of *r*-e5′NT Site-Finder utility of MOE was used. The docking of selected compounds within the active site of protein was carried out using the Triangular Matching docking method and 30 conformations of each ligand–protein complex were generated based on binding free energies. Base on free binding energy values, poses were sorted out and poses having lowest binding energy values was considered as the most stable ones with highest affinity for interaction with the receptor. Discovery studio visualizer v4 was used for analyzing the putative binding interaction with the side chains of amino acid residues in the active pockets of target enzymes.^35^

#### 2.2.5 DFT method

Computational DFT studies of eight compounds were performed by using SCM-ADF modeling suit 2018 installed on RCMS supercomputer. Molecular geometries of the investigated compounds were optimized using local density approximation (LDA) and GGA: PBE functional, core type medium and TZP basis set to get maximum accuracy. Based on geometry optimization at the above level of theory, Frontier molecular orbitals (HOMO, LUMO), band gap and molecular electrostatic potential (MEP) were also computed at GGA: PBE functional and TZP basis set to determine charge transfer behavior of the molecule^36^

## 3. Results and discussion

### 3.1 Chemistry

**Scheme 1:**
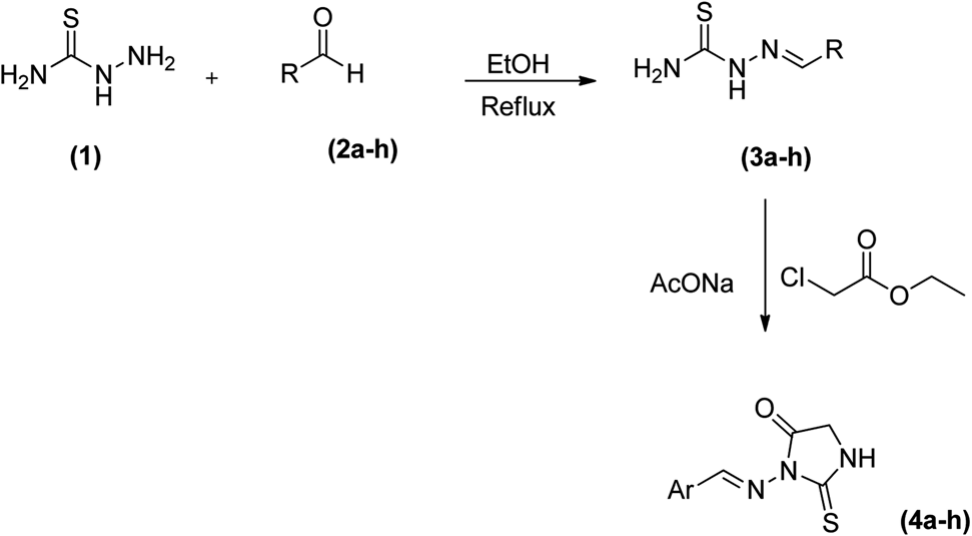
Synthesis route adopted for azomethine–Thioxoimidazolidinone conjugates (4a–h).

Thiosemicarbazide (1) was reacted with suitably substituted aldehyde in dry ethanol under reflux conditions to afford solid products which were purified by recrystallization using ethanol to give thiosemicarbazones (3a–h). Reaction of the latter (3a–h) with an equimolar amount of ethyl chloroacetate in the ethanol in the presence of fused sodium acetate followed by recrystallization from ethanol afforded (4a–h) in excellent yields (Scheme 1).

### 3.2 Structure activity relationship

**Scheme 2:**
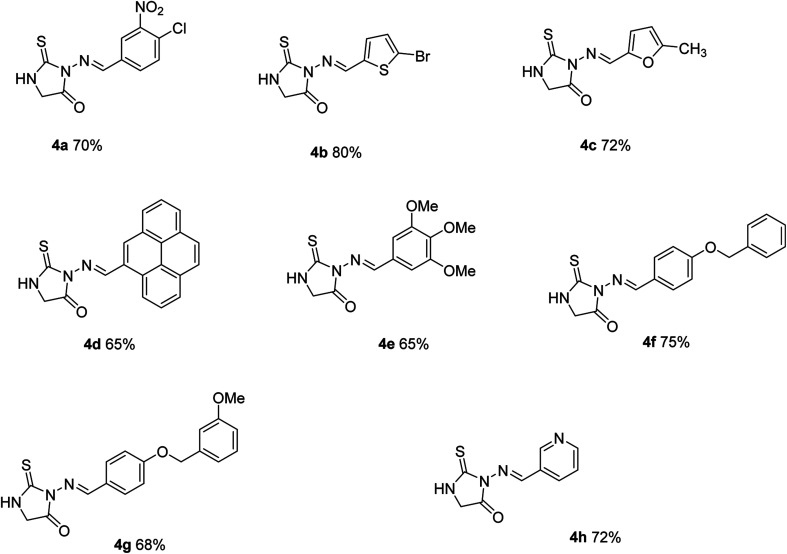
Chemical structures of azomethine–Thioxoimidazolidinone conjugates (4a–h).

The synthesized compounds were tested for their inhibitory potential against both human and rat e-5′NT activity. These derivatives possessed different aromatic substituent with their inhibitory value in the range of 0.18 to 42.8 μM and 0.24 to 57.6 μM against human and rat e5′NT, respectively (Table 1). All derivatives displayed inhibition against *h*-e5′NT whereas compound 4e showed maximum potential with IC_50_ values 0.18 ± 0.05 μM. The detailed study of this derivative revealed that it possessed sterically hindered 1,2,3-trimethoxyphenyl ring. The presence of methoxyl groups (–OCH_3_) increased the electron density of phenyl ring thus, possibly increased its reactivity towards the enzyme and therefore, responsible for the maximum inhibition. Similarly, in case of derivative 4g, the attachment of substituent 3-(phenoxymethyl)phenol resulted in comparable inhibition potential (IC_50_ ± SEM = 0.23 ± 0.08 μM) as compared to compound 4e. The substitution effect of 1-chloro-2-nitrobenzene group (4a) resulted in significant loss of inhibitory potential (IC_50_ ± SEM = 4.12 ± 0.12 μM). It might be due to the decreased reactivity of phenyl ring with substitution of electron withdrawing group such as nitro and chloro-group. The thioxoimidazolidine derivatives possessing aromatic ring other than benzene (4b, 4c, 4h) were less potent than derivative 4e and 4g. Among them, the derivative (4b) containing 2-bromo-thiphene ring substituent was more active with IC_50_ ± SEM = 2.69 ± 0.06 μM value as compared to other derivatives (4c and 4h). The thiophene ring possessed greater aromatic behavior along with greater reactivity against its target than other aromatic substituent. Substituent such as furan (4c) and pyridine ring (4h) exhibited less inhibitory effect with IC_50_ ± SEM 11.2 ± 0.32 and 42.8 ± 0.64 μM, respectively. Another derivative 4d displayed less inhibitory behavior with IC_50_ ± SEM = 4.17 ± 0.17 μM. When these derivatives were tested against rat isozymes it displayed different inhibitory behavior. Among all derivatives, two derivatives (4d, 4g) remained inactive while three derivatives (4a, 4b, 4e) showed good inhibition. The derivative 4e displayed maximum inhibition towards rat enzyme with IC_50_ ± SEM = 0.24 ± 0.03 μM which is nearly 322-fold higher than the control inhibitor sulfamic acid. The derivative 4a showed not as much of inhibition (IC_50_ ± SEM = 1.03 ± 0.11 μM) as compared to compound 4e that might be due to the substitutional effect of 1-chloro-2-nitrobenzene group. The replacement of phenyl ring substitution with 2-bromo-thiophene ring (4b) resulted in comparable inhibition with IC_50_ ± SEM = 0.37 ± 0.06 μM as compared to 4e. Other derivatives such as 4c and 4h exhibited less inhibitory activity with IC_50_ ± SEM values 57.6 and 19.7 μM respectively. Hence, the phenyl ring substitution resulted in greater inhibitory behavior against both *h*-e5′NT and *r*-e5′NT enzyme. The previously identified potent compound of our lab *i.e*., sulfamic acid is used as positive control as there is currently no drug available in market as CD73 inhibitor. Although, some ADP analogue molecules are presently in clinical trial stages (Scheme 2).

**Table 1:** Ecto-5′-nucleotidase (*h*-e5′NT and *r*-e5′NT) inhibition and cytotoxic potential of (3-(aryl)amino)-2-thioxoimidazolidin-4-one derivatives

Compound	Enzyme inhibition data		Cytotoxic potential	
	*h*-e5′NT	*r*-e5′NT	HeLa	BHK-21
	μM ± SEM		% inhibition value	
(4a)	4.12 ± 0.12^a^	1.03 ± 0.11^a^	90.2%^b^	4.31%^b^
(4b)	2.69 ± 0.06^a^	0.37 ± 0.06^a^	84.3%^b^	2.57%^b^
(4c)	11.2 ± 0.32^a^	57.6 ± 1.27^a^	87.0%^b^	6.72%^b^
(4d)	4.17 ± 0.17^a^	31.4%^b^	72.1%^b^	3.88%^b^
(4e)	0.18 ± 0.05^a^	0.27 ± 0.03^a^	60.0%^b^	5.78%^b^
(4g)	0.28 ± 0.08^a^	42.5%^b^	71.3%^b^	10.2%^b^
(4h)	42.8 ± 0.64^a^	19.7 ± 0.98^a^	82.2%^b^	12.6%^b^
Sulfamic acid	42.1 ± 7.8^a^	77.3 ± 7.0^a^	—	—
Carboplatin	—	—	85.2%^b^	18.4%^b^

a The IC_50_ value shows the concentration of tested compound required to inhibit 50% enzyme level (used during assay).

b Represents the percent inhibition value. The experiment was performed in triplet (*n*) and results are given with standard error of mean (S.E.M) which shows the reliability of results and assay sensitivity.

### 3.3 Cytotoxic potential (cell viability assay, MTT)

The cytotoxic potential of synthesized derivatives was tested against the target cell line having higher expression of e5′NT enzyme and also against the normal cell line BHK-21. The obtained results have been mentioned in Table 1. It was found from Table 1 that all these derivatives exhibited cytotoxic potential against the cervical cancer cell line while no activity against the normal one *i.e.*, BHK-21. These derivatives exhibited cytotoxic potential in the range of 60 to 90% against the HeLa cell line at final concentration of 100 μM. In present experiment, the carboplatin was used as reference standard having cytotoxic potential of 85% at 100 μM. All derivatives were tested at 50 μm concentration and their potential was determined after 24 h. Overall, the synthesized derivatives that showed non-selective enzymatic activity against human and rat e5′NT also displayed better cytotoxic potential (80–90%) against cervical cancer cell line. The other two derivatives possessing selective inhibition data against human e5′NT exhibited somewhat lower cytotoxic potential *i.e.*, 70%.

### 3.4 CD73 mRNA expression analysis using qRT-PCR

**Fig. 1:**
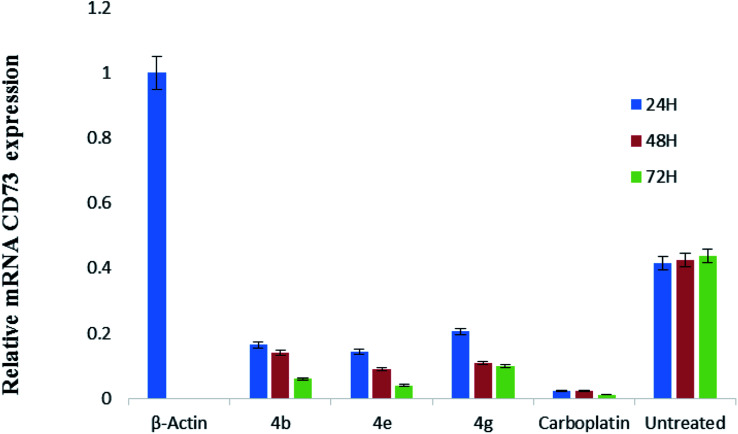
Relative mRNA expression of CD73. The graph showing the reduced expression of CD73 in comparison with control (β-actin) after treating with the compound 4b, 4e and 4g at 24, 48 and 72 h.

CD73 represent a vital function in the modulation of the action of adenosine in the progression of tumor. This nucleoside, by sensitizing adenosine receptors, can favor tumor growth and stimulate angiogenesis, immune response suppression and cell proliferation. So, this enzyme is involved in over expression of cancer cells. Cancer cells (HeLa cell line) was treated with the most potent compounds 4b, 4e and 4g for different time interval *i.e.*, 24, 48 and 72 h and measured the expression level of CD73 *via* relative quantification RT-PCR method. Our results found that relative mRNA expression of CD73 was 83% downregulated after 24 h and 95% down-regulation was observed after 72 h treatment with compound 4b, while compound 4g showed 80 to 85% down-regulation. Compound 4e showed 88% down-regulation after 24 h and 96% down-regulation was observed after 72 h of treatment in comparison with positive control (β-actin). Carboplatin significantly reduced the CD73 expression after 24, 48 and 72 h treatment as shown in Fig. 1. Untreated cervical cancer cells showed increased up-regulation of CD73.

### 3.5 Molecular docking studies

**Fig. 2:**
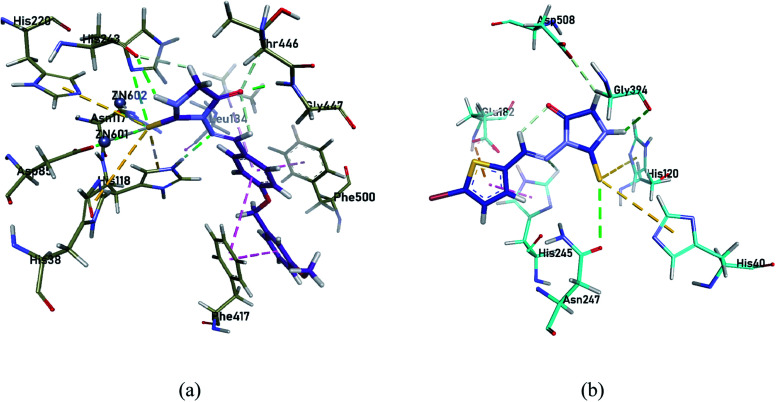
Compound 4g inside the active pocket of h-e5′NT (a) and compound 4b inside the active pocket of r-e5′NT (b).

Molecular docking studies for the most potent selective inhibitors were performed to assess the molecular interactions between the ligands and the enzymes. As suggested by the *in vitro* results, 4g compound exhibited the profound selective inhibition for *h*-e5′NT while 4b showed the most potent selective inhibition for *r*-e5′NT enzyme. The amino group of thioxoimidazolidinone ring formed hydrogen bond with Asp85, Asn117, His118, His243, Thr446 and Gly447 residues of the protein, while alkyl groups of hybrid displayed π–π interactions with Leu184, Phe417 and Phe500 residues to collaborate in enzyme inhibition. The two Zn^2+^ ions in the active pocket of the protein were also seen to chemically interact with the sulfur group of thioxoimidazolidinone of compound 4g (Fig. 2a). For *r*-e5′NTenzyme, the compound 4b was found the most potent selective inhibitor. Sulfur and amino group of thioxoimidazolidinone ring exhibited hydrogen bonding with His245, Asn247, Gly394 and Asp508 residues of the protein while sulfur group from thiophene ring formed π–sulfur interactions with His40, His120 and Glu182 residues of the protein (Fig. 2b). These interactions were sufficing to correlate the inhibition of the enzymes with these compounds as indicated in the *in vitro* analysis.

### 3.6 Computational structure analysis by DFT studies (4a–h)

**Fig. 3:**
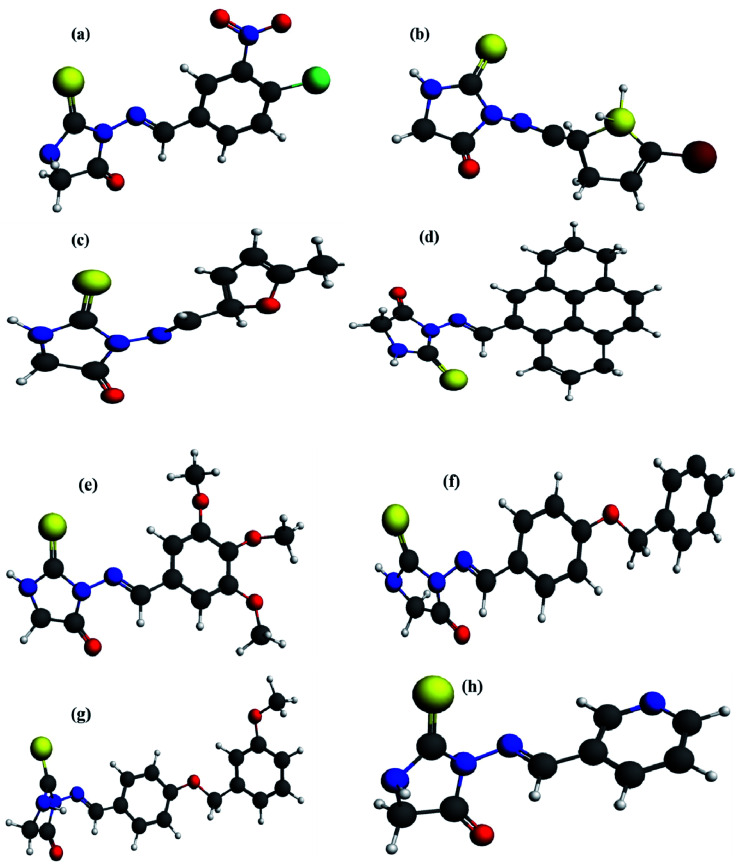
Quantum chemically optimized geometries of compounds (4a–h) using DFT at GGA-PBE functional and TZP basis set with numerical accuracy of 3.0.

**Fig. 4:**
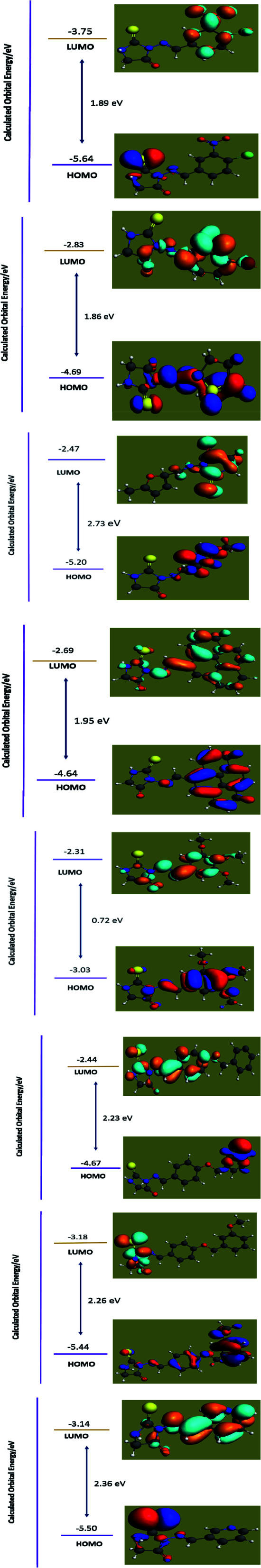
Frontier molecular orbital energy gap b/w HOMO and LUMO of compound (4a–h) from top to bottom at GGA-PBE functional and TZP basis set with numerical accuracy of 3.0.

**Fig. 5:**
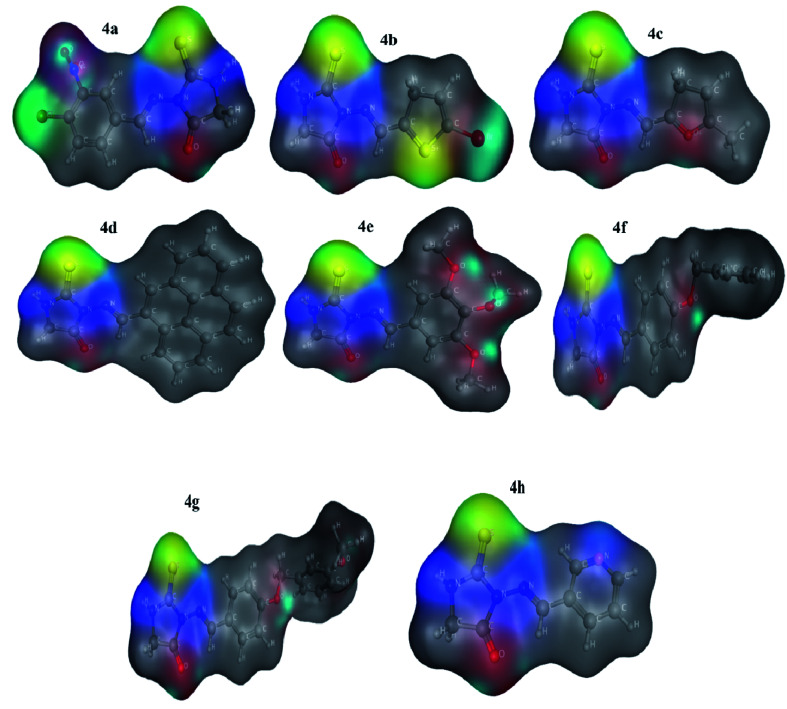
Molecular electrostatic potential surfaces (MESP) of compounds determined at LDA GGA: PBE using TZP basis set.

Quantum chemical DFT investigations have substantial importance to illustrate the electronic structure, charge distribution and evaluation of related parameters. Herein; Optimized geometries, HOMO-orbital, LUMO-orbital and their respective frontier orbital energies (*E*_HOMO_ and *E*_LUMO_), and surface electrostatic potential map are shown in Fig. 3–5 respectively. Optimized structures of compounds (4a–h) have shown to have non-planner geometries which have also been depicted in molecular docking studies as well. Energies of frontier molecular orbitals (FMOs), *i.e.*, *E*_HOMO_ and *E*_LUMO_, provide an estimate of electron-donating and electron-accepting nature of a molecular system. *E*_HOMO_ and *E*_LUMO_ gap (Δ*E*) determine the kinetic stability of a molecular system, chemical reactivity, and resistance to change in electronic distribution of a molecule.^37^Fig. 4 reveals lowest Δ*E* value for 4e determing its higher reactivity and electron transfer capability as compared to other compounds . A *E*_HOMO_–*E*_LUMO_ energy gap has inverse relationship with the binding tendency of an investigated compound, hence 4e is greater binding affinity.^38^Fig. 4 indicated Δ*E* as 1.8878 eV, 1.8594 eV, 0.7184 eV, 1.9485 eV, 2.7333 eV, 2.2287 eV, 2.2631 eV, 2.3591 eV respectively. Lesser HOMO–LUMO energy gap (Δ*E*) indicated to have a greater tendency of electronic overlap with enzymes due to presence of 1,2,3-trimethoxyphenyl ring and inferred to have greater binding strength. Compound 4c and 4h was found to have highest Δ*E* and lowest reactivity due to maximum electronic distribution of LUMO on methyl pyridyl ring and pyridyl ring respectively. Due to higher density distribution at LUMO, tendency of electron transfer from HOMO is restricted thus reducing it reactivity for interactions with enzyme.

### 3.7 Molecular electrostatic potential analysis

The MESP which is measure of the electronic density distribution is a very valuable property to describe electrophilic and nucleophilic centers in a chemical compound. It also responsible to furnish hydrogen-bonding interactions between the molecules.^39^ Molecular Electrostatic Potential Surfaces (MESP) of optimized geometries of eight compounds at GGA: PBE level of theory was determined to map the electrophilic and nucleophilic regions of compounds as indicated in Fig. 5. It is apparent from Fig. 3 that negative potential is concentrated on oxygen (red), hence representing nucleophilic character to O atoms with molecular structure. A positive potential is strenuous on C as well as H atoms depicting their electrophilic nature while taking part in a chemical process. Negative and positive potential regions give us information about the sites involved in intermolecular interactions denoting –O atom as having electron donating character in compounds. Dispersion of potentials for titled compounds was found to be ranging from −0.0853 to 0.0853 esu.

Electrostatic potential maps of compounds (4a–h) have shown the high negative charge density on two nitrogen atoms and less negative charge density on S atom of the optimized compounds and hence S atom has a greater possibility to develop interactions with electron rich amino acids of enzyme, resulting in the stability of compound–enzyme complex.

## 4. Conclusion

A small set of azomethine–thioxoimidazolidin derivatives was examined for their enzyme inhibition potential against both human and rat isozyme was evaluated. These derivatives displayed significant inhibitory potential against *h*-e5′NT as compared to *r*-e5′NT. They also possessed significant anticancer potential against different cancer cell lines such as, MCF-7 and HeLa cells. Compound 4g displayed selective and potential inhibition towards *h*-e5′NT with IC_50_ value of 0.23 ± 0.08 μM. The compound 4h displayed nonselective as well as potent enzyme inhibition against both human and rat e5′NT. Compound 4e downregulated the CD73 expression level up to 95%. DFT studies were performed at LDA-GGA level of theory which revealed that *E*_HOMO_–*E*_LUMO_ gap (Δ*E*) determined higher reactivity of 4e. MESP potential surface analysis indicated electrophilic nature of –S atom in compounds for their interactions with enzyme residues. Moreover, molecular docking studies were also performed to determine their putative binding sites. Hence, these derivatives can be further evaluated for their therapeutic importance in the management of various diseases.

## Conflicts of interest

There are no conflicts to declare.

## Supplementary Material

RA-012-D2RA02675A-s001
