# Supplementary material for: Appraisal of novel azomethine–thioxoimidazolidinone conjugates as ecto-5′-nucleotidase inhibitors: synthesis and molecular docking studies
Source: RSC Adv. 2022 Jun 14;12(27):17596–606. doi: 10.1039/d2ra02675a (PMC9194930; doi:10.1039/d2ra02675a)
Supplement: RA-012-D2RA02675A-s001 [file RA-012-D2RA02675A-s001.pdf]

Electronic Supplementary Material (ESI) for RSC Advances.
This journal is © The Royal Society of Chemistry 2022

Supporting Information

Appraisal of Novel Azomethine-Thioxoimidazolidinone conjugates as ecto-5′-nucleotidase inhibitors: Synthesis and Molecular Docking Studies

Pervaiz Ali Channar[a,b†], Sehrish Bano[c†], Sidra Hassan[c], Fouzia Perveen[d], Aamer Saeed[a*], Parvez Ali Mahesar[e], Imtiaz Ali Khan[f], Jamshed Iqbal[c*]

[a]Department of Chemistry, Quaid-I-Azam University, 45320, Islamabad, Pakistan

[b]Department of Basic sciences, Mathematics and Humanities, Dawood university of Engineering and Technology, Karachi74800, Pakistan

[c]Centre for Advanced Drug Research, COMSATS University Islamabad, Abbottabad Campus, Abbottabad-22060, Pakistan

[d]Research Center for Modeling and Simulations, National University of Sciences and Technology (NUST), Islamabad, Pakistan

[e]Institute of Chemistry, Shah Abdul Latif University, Khairpur, 66020, Pakistan

[f]Department of Entomology, Agricultural University, Peshawar 25130, Khyber Pakhtunkhwa, Pakistan.

[†] Both these authors contributed equally

[*]**Corresponding Authors**:

Prof. Dr. Aamer Saeed; aamersaeed@yahoo.com, asaeed@qau.edu.pk

Prof. Dr. Jamshed Iqbal; drjamshed@cuiatd.edu.pk

Supplementary

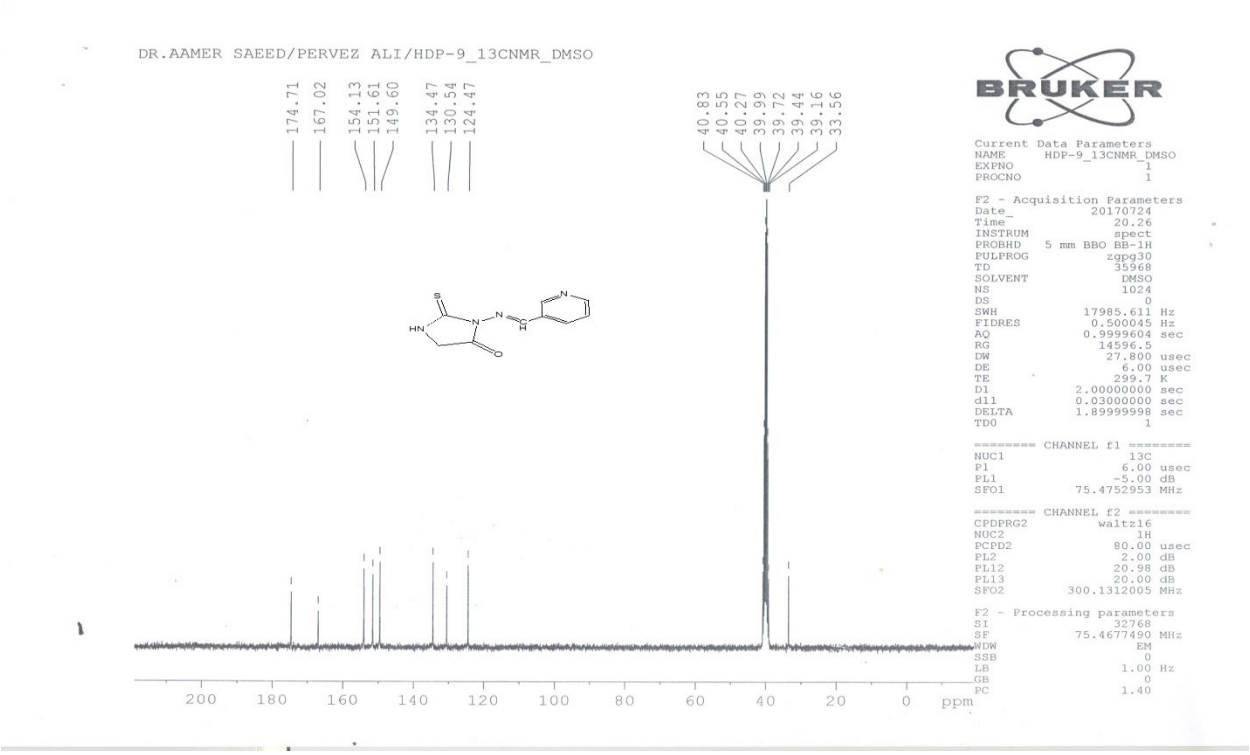

(*E*)-3-((pyridin-3-ylmethylene)amino)-2-thioxoimidazolidin-4-one^{13}CNMR (4h)

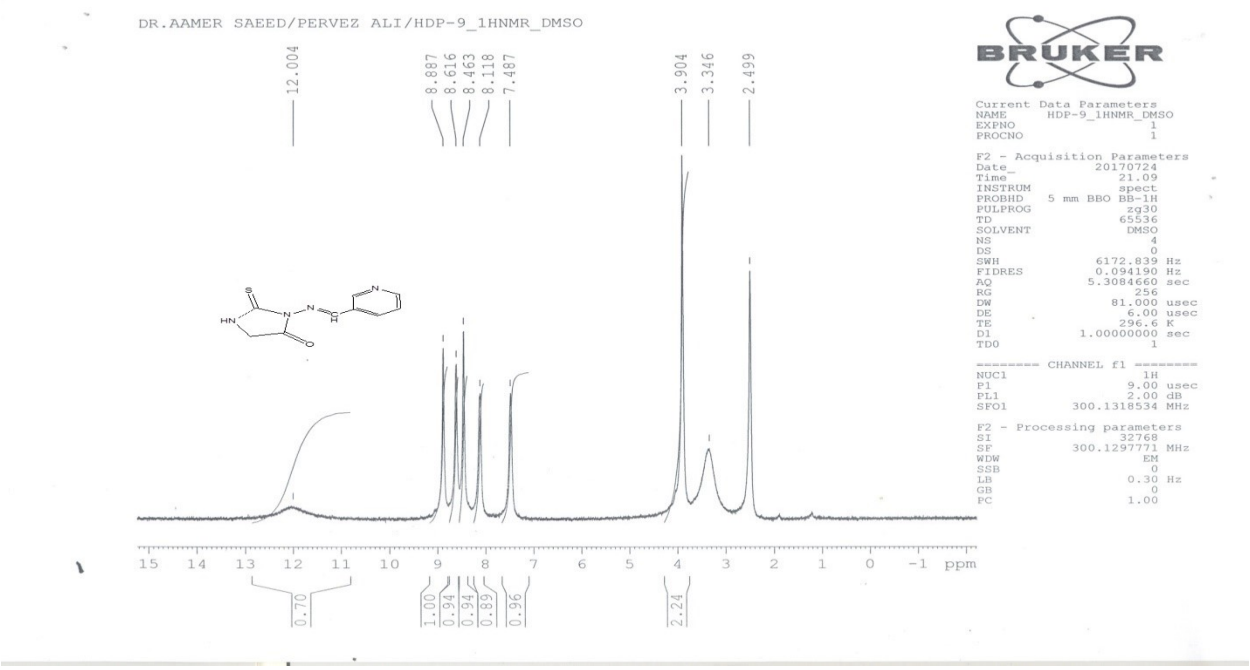

(*E*)-3-((pyridin-3-ylmethylene) amino)-2-thioxoimidazolidin-4-one^{1}HNMR (4h)

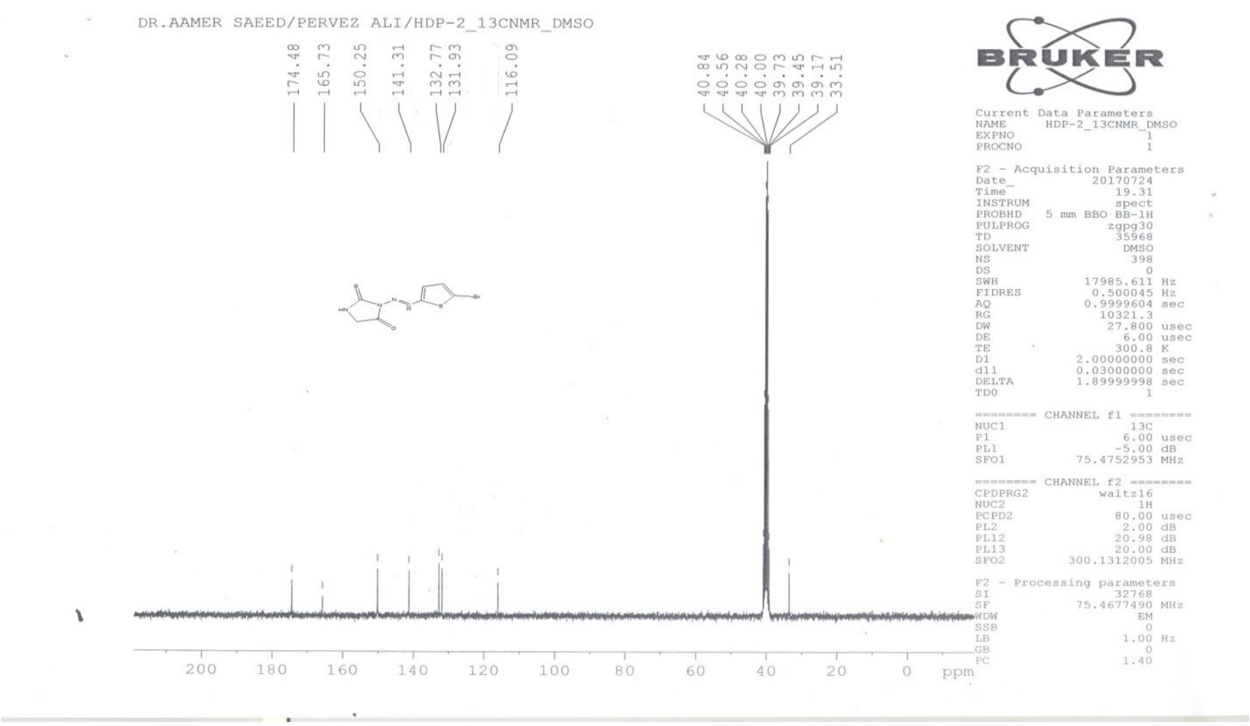

(*E*)-3-(((5-bromothiophen-2-yl)methylene)amino)-2-thioxoimidazolidin-4-one^{1}HNMR(4b)

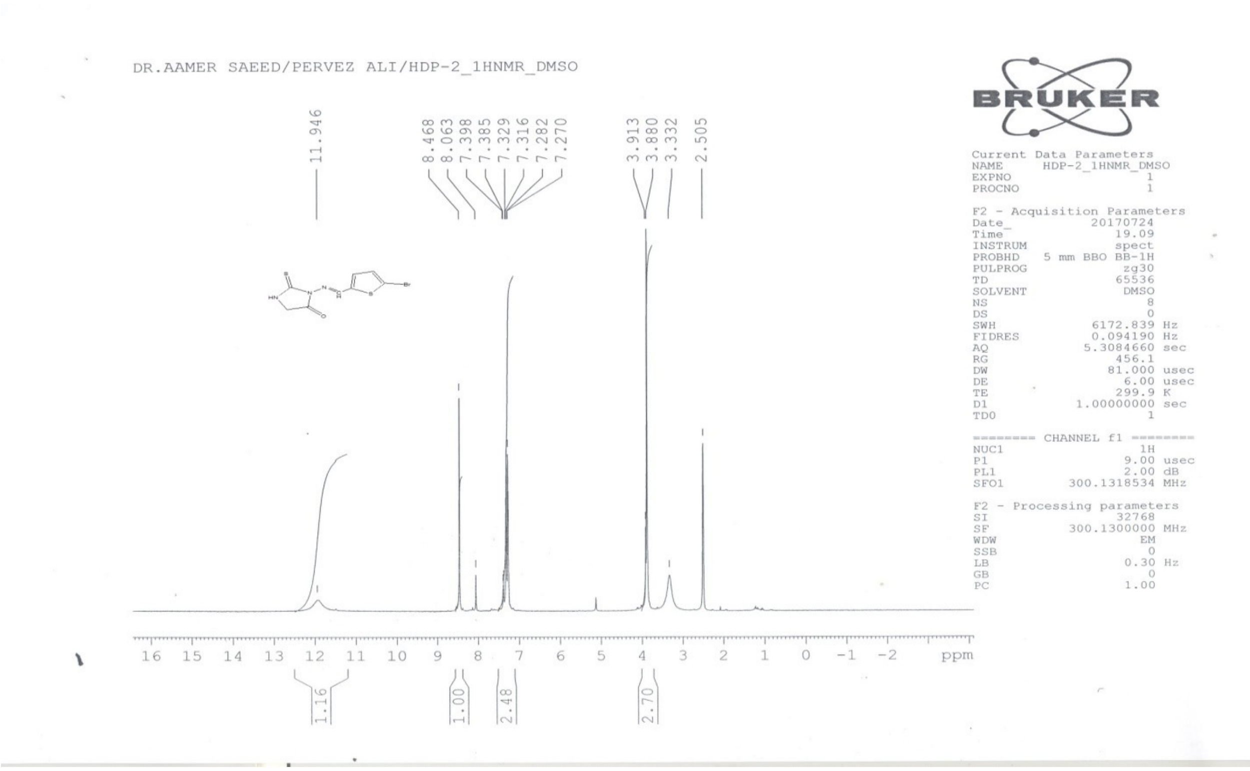

(*E*)-3-(((5-bromothiophen-2-yl)methylene)amino)-2-thioxoimidazolidin-4-one^{13}CNMR (4b)

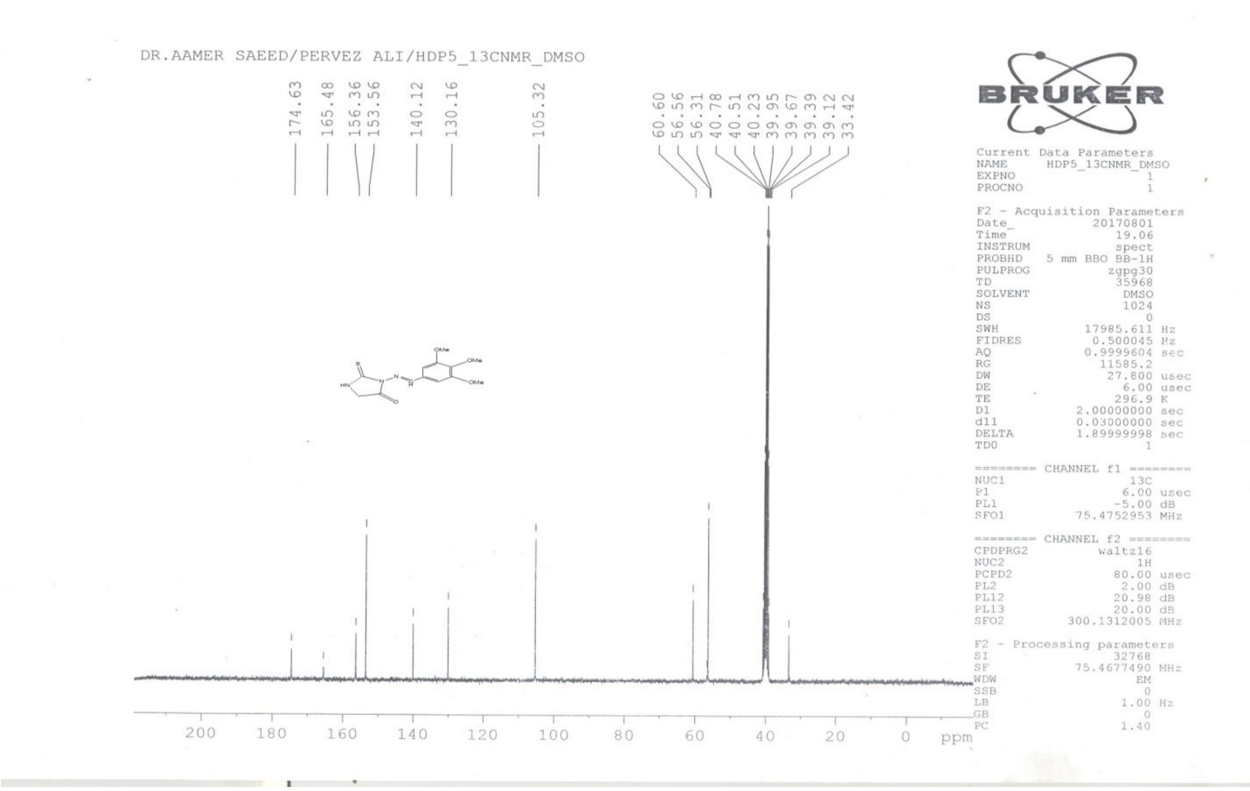

(*E*)-2-thioxo-3-((3,4,5-trimethoxybenzylidene)amino)imidazolidin-4-one^{1}HNMR (4e)

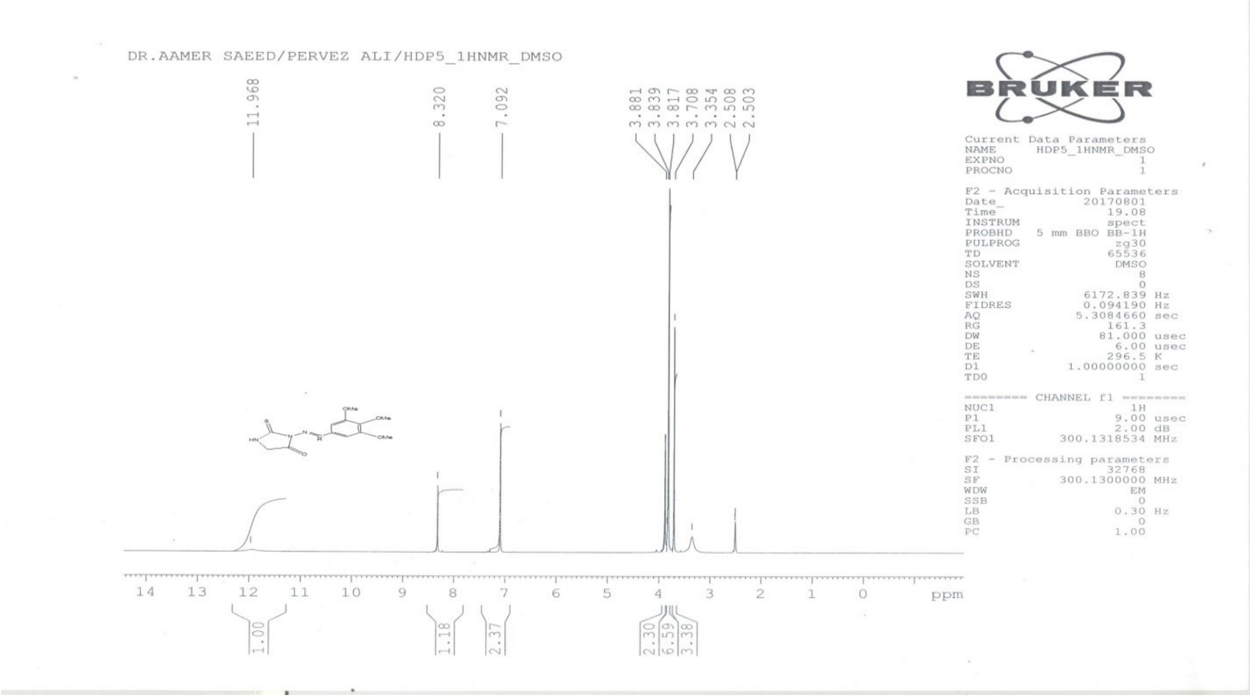

(*E*)-2-thioxo-3-((3,4,5-trimethoxybenzylidene)amino)imidazolidin-4-one^{13}CNMR (4e)
